# Augmented Prediction of N Parameter in Breast Cancer: Is It Possible with Shear-Wave Elastography Ultrasound Radiomics?

**DOI:** 10.3390/cancers18050862

**Published:** 2026-03-07

**Authors:** Martina Caruso, Ludovica Rita La Rocca, Arnaldo Stanzione, Nicola Rocco, Tommaso Pellegrino, Daniela Russo, Maria Salatiello, Andrea de Giorgio, Roberta Pastore, Simone Maurea, Arturo Brunetti, Renato Cuocolo, Valeria Romeo

**Affiliations:** 1 Department of Advanced Biomedical Sciences, University of Naples Federico II, 80138 Naples, Italy; martina.caruso@unina.it (M.C.); arnaldo.stanzione@unina.it (A.S.); nicolarocco2003@gmail.com (N.R.); danielarusso83@yahoo.it (D.R.); maurea@unina.it (S.M.); brunetti@unina.it (A.B.); 2 Azienda Ospedaliera Universitaria Federico II, Via S. Pansini 5, 80131 Naples, Italy; ludovicarita.larocca@gmail.com (L.R.L.R.); tompellgrino@hotmail.com (T.P.); m.salatiello@gmail.com (M.S.); robpast@libero.it (R.P.); 3 Artificial Engineering, Via del Rione Sirignano, 80121 Naples, Italy; andrea@degiorgio.info; 4 Department of Medicine, Surgery and Dentistry, University of Salerno, 84081 Baronissi, Italy; renato.cuocolo@gmail.com

**Keywords:** artificial intelligence, breast cancer, machine learning, N parameter, axillary lymph node status, radiomics

## Abstract

**Background/Objectives:** The aim was to assess whether a machine learning (ML) algorithm could empower the ability of ultrasound (US) integrated with shear-wave elastography (SWE) to preoperatively define the ALN status in breast cancer (BC). **Methods:** Patients with at least one histologically proven BC lesion, who underwent preoperative breast US and SWE were retrospectively enrolled. BC lesions were segmented on US and SWE images by three different operators and radiomics features were extracted. A multi-step US and SWE feature selection was performed. A Simple Logistic ML classifier was applied to the dataset to predict the ALN status, its performance assessed through the AUC and Matthews Correlation Coefficient (MCC). The performance of the ML classifier was compared to that of an expert radiologist, who evaluated the US B-mode lymph-node features included in the test set. **Results:** A total of 133 BC lesions were included and divided into a training set, composed of 89 BC lesions (ALN−: 52; ALN+: 37), and a test set, including 44 BC lesions (ALN−: 24; ALN+: 20). Eight features out of the 1098 radiomics features extracted from US and SWE images were selected to build the predictive model. Simple Logistic classifier showed AUC of 0.685 and 0.677, MCC of 0.387 and 0.375 in the training and test set, respectively. The performance of the expert radiologist was higher than that of the ML classifier (AUC = 0.817), but not significantly different (*p* = 0.481). **Conclusions:** The inclusion of SWE-derived radiomics features could aid in the preoperative assessment of ALN status in BC using an ML approach.

## Simple Summary

Ultrasound (US) is still the most sensitive modality to predict axillary lymph node (ALN) status in patients with breast cancer (BC) but suffers from a low and variable specificity. A Simple Logistic Machine Learning algorithm was used with US B-mode and SWE-derived radiomics features of 133 primary BC lesions to identify cases with positive ALN status. The classifier showed AUC of 0.685 and 0.677, MCC of 0.387 and 0.375 in the training and test set, respectively. The performance of ML was lower, even if not significantly (*p* = 0.481) from that of an expert radiologist (AUC = 0.817) who evaluated US images of ALN in the test set. Although the accuracy of ML was relatively low compared to the values reported in the literature, our findings support the inclusion of SWE-derived radiomics features of the primary BC lesion in a radiomics pipeline for the prediction of ALN status.

## 1. Introduction

The axillary lymph node (ALN) status represents one of the most important independent prognostic factors for predicting disease-free survival and overall survival in patients with breast cancer (BC), because it reflects the risk of distant recurrence and death after loco-regional treatment [1]. The 5-year survival rate drops from 98.6% to 84.4% when ALN positivity is present at diagnosis [2]. Therefore, accurate assessment of ALN metastases is critical for the clinical decision-making process. Currently, the gold standard for axillary staging in patients with BC is the histological examination of sentinel lymph-node biopsy (SLNB) and/or axillary lymph-node dissection (ALND). Unfortunately, both procedures are invasive and have a high incidence of complications, such as lymphedema, upper limb neuropathy, and limited movement of the shoulder [3,4]. In current clinical practice, ALN status can be preoperatively assessed using different imaging techniques, such as US, magnetic resonance imaging (MRI) and 18F-FDG Positron Emission Tomography/Computed Tomography (PET/CT) [5]. Among these, ultrasound (US) plays a leading role, being widely available, easy to perform, cost effective, and able to guide biopsy. This technique has high sensitivity (87%) in assessing ALN status but still suffers from low and variable specificity (53–97%) due to high operator-dependence, while providing qualitative information [6]. The clinical implications of US assessment of ALN status have become even more relevant in light of the evidence from the SOUND (Sentinel Node vs. Observation after Axillary Ultrasound) randomized clinical trial. Indeed, it demonstrated that omitting axillary surgery was noninferior to SLNB in patients with BC up to 2 cm and a negative axilla on US [7,8]. Therefore, the development of a noninvasive ALN staging method to accurately assess BC clinical stage and select tailored treatment options for patients is an urgent need. In this scenario, a relatively new technique, breast shear-wave elastography (SWE), has been introduced to enhance the US diagnostic accuracy in characterizing breast lesions and, consequently, improve patient management [9,10]. It provides qualitative data, shown as a semitransparent color-coded image, and quantitative data, expressed as shear wave velocity (m/sec) or elasticity (kPa) measured within a specified region of interest (RO [11]. The potential enhancing role of SWE in predicting ALN status in patients with BC has not been investigated to date [12,13].

Over the last few years, the possibility of extracting quantitative data from medical images, so-called radiomics, has opened new research perspectives for detecting tumor features invisible to the human eye and possibly correlated with cancer heterogeneity and behavior, using artificial intelligence (AI) software [14,15]. Regarding the potential applications of radiomics and AI in the diagnosis and management of BC, several investigations have used US, digital breast mammography, or MRI with different aims, including differential diagnosis of breast lesions, prediction of BC molecular subtypes, and assessment of ALN status [16,17,18]. In detail, regarding this latter task, most machine learning (ML) models were built using quantitative parameters extracted from primary tumor lesions on MR image [19,20].

### Objective of the Study

The aim of this study was to assess whether an ML algorithm incorporating radiomic features extracted from US B-mode and SWE images of the primary breast lesion could enhance the ability of US to preoperatively define ALN status in BC patients. The goal was to further improve the diagnostic accuracy of US, which is currently the most sensitive modality for this purpose.

## 2. Materials and Methods

This retrospective study was approved by the local Ethic Committee of the *BLINDED* and written informed consent was waived. The study was conducted in accordance with the CLEAR checklist (Checklist for Laboratory Evidence And Reporting) and the METRICS guidelines (Minimum Information for Reporting a Radiomics Study) to ensure transparency and reproducibility in radiomics pipeline [21].

In detail, the CLEAR checklist is a structured assessment tool designed to enhance transparency, methodological quality, and reproducibility in radiomics studies. It provides standardized criteria for reporting key aspects of radiomics workflows, including image acquisition, feature extraction, data processing, model development, and validation. The METRICS guidelines constitute a methodological framework for the construction and evaluation of radiomics models, focusing on robust feature selection, model validation, and reliable performance assessment.

### 2.1. Patient Population

Breast US examinations integrated with SWE performed between January 2021 and July 2023 at *BLINDED* were retrospectively reviewed. Clinical indications for US included routine check-up, assessment of a palpable mass, and mammographically suspicious findings. US examinations were performed by a radiologist with 10 years of experience in breast imaging. The inclusion criteria were as follows: >18-year-old patients with histologically proven BC for whom ALN status was available, in terms of biopsy of a clinically suspected ALN, SLNB, or ALND. The exclusion criteria were as follows: US and/or SWE images not available and/or suitable for the subsequent analysis (i.e., images in which the lesion was not fully included, or affected by motion artifact); and not available histological reports. The following clinical data were collected for each patient: age at diagnosis, lesion size (maximum diameter), histological data for the BC lesion (tumor grade, histologic subtype, and molecular subtype), and ALN status (positive or negative for micrometastasis or macrometastasis).

### 2.2. Standard of Reference

The ALN status was assessed through the biopsy of a clinically suspected ALN in patients who were candidates for neoadjuvant systemic treatment, while SLNB or ALND were considered for patients who underwent upfront surgery.

### 2.3. Image Acquisition

US examinations were performed using a LOGIQ S8, GE Healthcare US scanner, employing a high-frequency linear probe (6–15 MHz) with radial, transverse, and longitudinal scans on both breasts. SWE was performed using a dedicated 9 MHz linear probe. Details of the US acquisition protocol are reported in Supplementary Materials S1.

### 2.4. Imaging Conversion and Segmentation

**Figure 1:**
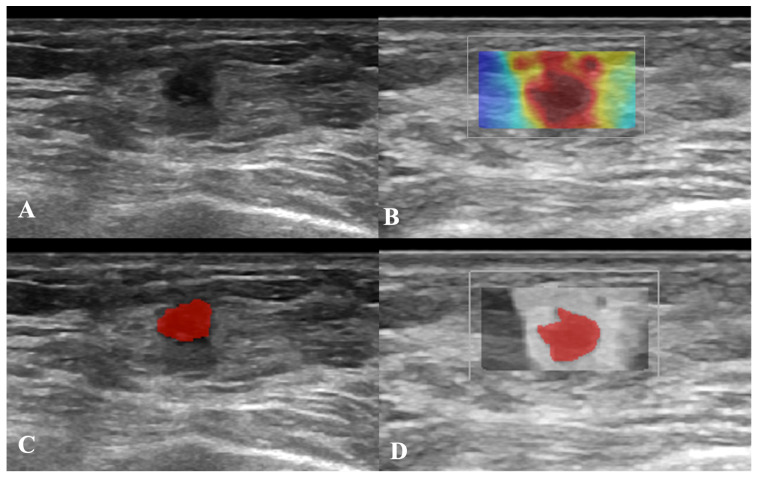
Example of breast lesion annotation. The first column shows manual placement of a region of interest on B-mode image (**A**,**C**), while the second one on SWE image (**B**,**D**).

After evaluation of US examinations by a dedicated breast radiologist, DICOM images in which the lesion was fully included, free of artifacts, and with measurements were selected and retrieved from the digital archives. Since US images were originally encoded as three-channel RGB images, an ITU-R 601-2 luma transform was applied to convert them to grayscale. Thereafter, a dedicated software (ITKSNAP, v3.8.0) was used to manually segment breast lesions on B-mode and SWE US images, yielding 2D regions of interest (ROIs) [22] Figure 1 shows an example of manual breast-lesion segmentation on B-mode (A and C) and SWE images (B and D). Furthermore, to assess feature stability, two independent senior radiology residents manually segmented 30 randomly selected B-mode and SWE US images from the training set. The feature stability was estimated using the intraclass correlation coefficient (ICC) [23]. Each operator paid particular attention to remaining within the lesion margins during manual segmentation.

### 2.5. Image Preprocessing and Feature Extraction

Image preprocessing and 2D features extraction were performed according to the Imaging Biomarker Standardization Initiative using a dedicated open-source Python-based software (PyRadiomics, v3.0.1) [24]. Image preprocessing and 2D features extraction were performed according to the Imaging Biomarker Standardization Initiative using a dedicated open-source Python-based software (PyRadiomics, v3.0.1) [24]. Pixels were resampled to 3 × 3 dimensions prior to feature extraction. Gray-level whole-image normalization was performed to ensure comparability across images acquired with varying settings, yielding a range of 0–600. As suggested by the developers, a fixed bin width (=3) was used for discretization. Furthermore, image filters, such as Laplacian of Gaussian (LoG, sigma = 3, 4, 5) and wavelet transformation (all high- and low-pass filter combinations on x and y planes) were applied to reduce image noise and highlight textural characteristics. Specifically, the LoG filter applies an image smoothing operation that enhances the structural edges within the ROI. In this context, the sigma value determines the level of detail in the output: lower values produce finer images, while higher values produce coarser ones [25]. Wavelet decompositions allow to remove low signal areas from the images (i.e., image smoothing and edge detection) using high- and low-pass filter combinations [26]. Regarding the feature classes, 2D shape, first-order, gray-level co-occurrence matrix (GLCM), gray-level run-length matrix, gray-level size zone matrix, and gray-level dependence matrix were extracted. Since image acquisition varied in terms of depth and zoom, only adimensional 2D shape features were included in the analysis to avoid bias. For the remaining classes, all available features were calculated, except GLCM sum average, as suggested by the PyRadiomics developers due to known redundancy with other GLCM parameters. Formulas and definitions of the extracted features can be found on the official documentation (https://pyradiomics.readthedocs.io/en/latest/features.html, accessed on 27 February 2026).

### 2.6. Data Processing

Before processing the data, a stratified random partition was performed into training (67%) and test (33%) sets. This was designed to prevent information leakage from the hold-out test set during data processing, feature selection, and model training.

To ensure the machine learning algorithm converges to optimal results, it is good practice to scale the data, which means applying a formula to map the feature values to a specific range. Specifically, the radiomics features extracted from US and SWE images were scaled using a MinMax scaler with a target range of 0 to 1. The scaler was fit exclusively on training data and used to transform the training and test sets independently. Data preprocessing was performed using the “scikit-learn” (v1.61) and “pandas” (v2.2) Python packages.

### 2.7. Data Analysis and Feature Selection

In the feature selection process, performed on training data, feature stability was tested by calculating a two-way random effect, single rater, absolute agreement ICC for each. Radiomics features with poor reproducibility (ICC value ≥ 0.75) were considered unstable and discarded in the following steps. Additionally, features with low variance (<0.01) and those highly correlated with each other (r > 0.80) in the Pearson test were excluded. Following these steps, the Synthetic Minority Oversampling Technique (SMOTE) was applied to the training data to balance the class samples [27,28]. In detail, SMOTE generates new instances (i.e., synthetic patients) for the minority class by interpolating data from the k nearest neighbors within the same label from the original population. This process was continued until the classes were fully balanced. Finally, the most informative variables were selected using an “information gain” ranking, with a threshold of 0.001. Feature selection was performed using the “irr” (v0.84.1) R package, the “scikit-learn” (v1.61) and “pandas” (v2.2) Python packages, as well as the WEKA (v3.7.10) platform.

### 2.8. Machine Learning Analysis

For the prediction of ALN status, Simple Logistic, an ML algorithm, was employed due to its computational efficiency, robust performance in binary classification tasks, and low risk of overfitting. The model’s performance was evaluated on the training set using stratified 10-fold cross-validation. This approach is more robust than a single partition split and is expected to provide a better estimate of the results’ generalizability [27]. In stratified cross-validation, each fold into which the data is divided preserves the class balance and serves as a validation set for an algorithm trained on the remaining (n = 9) folds. Then, the final model was fitted on the entire training set and evaluated on the held-out test set. The analysis was carried out using the WEKA software (version 3.7.10).

### 2.9. Radiological Evaluation

A dedicated breast radiologist performed a blinded assessment of the B-mode lymph node images, independent of the final diagnosis. Each lymph node was classified as positive or negative according to US B-mode features, such as shape, echo pattern, loss of central fatty hilum or thin hilum, eccentric thickening of the cortex, presence of microcalcifications, and ill-defined capsular margins [29]. To assess the potential added value of ML in improving radiologists’ performance, the operator was then asked to confirm or change the final diagnosis with the availability of ML readings.

### 2.10. Statistical Analysis

The Shapiro–Wilk test was first performed to assess whether data were normally distributed. Accordingly, Mann–Whitney U test was performed to assess differences in terms of age and lesion size (maximum diameter) between patients of the training and test sets. The ML classifier’s performance on the test set was compared to that of an expert radiologist using the McNemar test. A *p*-value < 0.05 was considered statistically significant. Statistical analysis was performed with dedicated software (IBM SPSS Statistics, Ver. 29.0.1.0, IBM Corporation, New York, NY, USA).

## 3. Results

### 3.1. Patient Population

A total of 213 patients were retrospectively enrolled according to the inclusion criteria. Then, 89 patients were excluded for lack of ALN status, and eight patients were excluded because US B-mode and/or SWE were not suitable for either radiomics or qualitative radiological evaluation. As a result, based on inclusion and exclusion criteria, the final study population included 116 patients (113 females, three males; mean age: 56.3 years, range 27–86 years) with a total of 133 breast lesions. Nine patients had multicenter or multifocal BCs with the same histologic subtype; of these, five patients had negative ALN status, while four had positive ALN status. Furthermore, six patients had bilateral BC. Mean size of breast lesions was 16.7 mm (range: 4–61 mm). Table 1 reports histological diagnosis and ALN status of the included breast lesions. The dataset was randomly split into a training set consisting of 89 breast lesions (67%), of which 52 had negative ALN status and 37 had positive ALN status, and a test set including 44 breast lesions (33%), of which 24 had negative ALN status, and 20 had positive ALN status. Age and lesion size were not statistically different between the two sets (*p* value of 0.479 and 0.805, respectively).

### 3.2. Machine Learning Analysis

**Figure 2:**
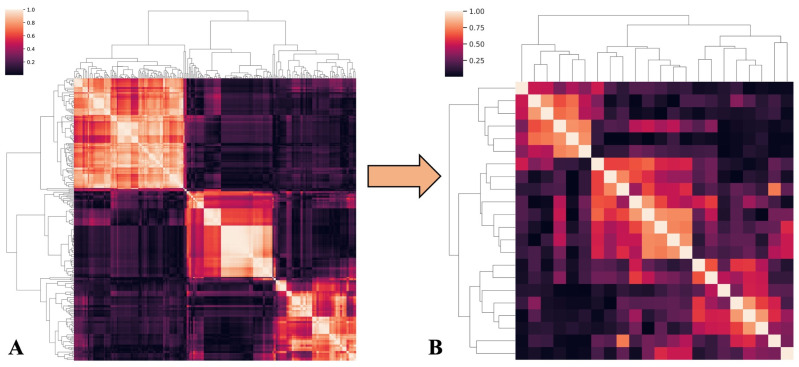
Clustermap of radiomics features correlation before (**A**) and after (**B**) selection with r > 80.

549 features were extracted from B-mode images and 549 from SWE images, with a total of 1098 features for each lesion. Of these, 835 were found to be unstable after ICC assessment and were discarded. None of the remaining 263 features showed low variance. Then, 241 features were excluded due to high pairwise correlations, leaving 22 features. Figure 2 shows a cluster map of radiomics feature correlations before (A) and after (B) selection. Panel B illustrates the dataset after removing features highly correlated with each other, applying r > 80.

After class balancing with SMOTE, eight of the remaining 22 features were selected through information gain ranking, including:

• bmode_original_glcm_JointEntropy;
• bmode_original_glcm-JointEnergy;
• bmode_original_firstorder_Energy;
• bmode_original_glrlm_GrayLevelNonUniformity;
• bmode_original_shape2D_PerimeterSurfaceRatio;
• swe_original_glrlm_GrayLevelNonUniformity;
• bmode_wavelet-H_glrlm_RunLengthNonUniformityNormalized;
• bmode_original_glrlm_ShortRunLowGrayLevelEmphasis.

**Figure 3:**
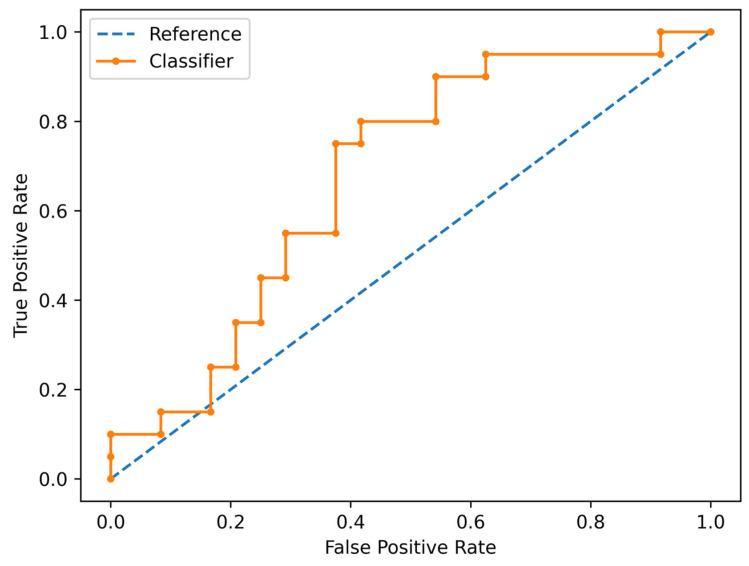
ROC curve of the Simple Logistic classifier in predicting ALN status.

**Figure 4:**
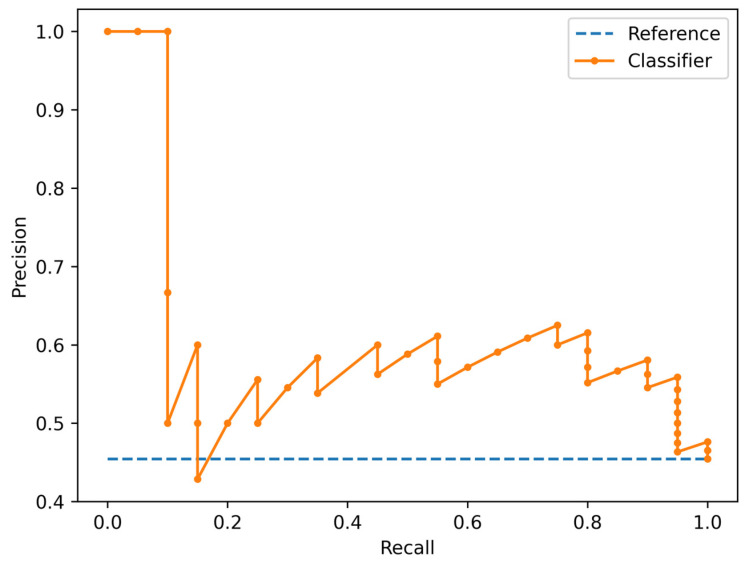
Precision Recall Curve showing the tradeoff between precision (positive predictive value) and recall (true positive rate) of the Simple Logistic classifier.

The Simple Logistic algorithm showed AUC values of 0.685 and 0.677 in the training and test sets, respectively, and a value of MCC (Matthews Correlation Coefficient) of 0.387 and 0.375 respectively. Specifically, it showed an accuracy of 68% (95% CI: 52–81%), sensitivity of 75% (95% CI: 51–91%), specificity of 63% (95% CI: 41–81%), positive likelihood ratio of 2.0 (95% CI: 1.1–3.6), negative likelihood ratio of 0.4 (95% CI: 0.2–0.9). ROC and Precision Recall Curve are shown in Figure 3 and Figure 4, respectively.

Table 2 reported the confusion matrix of the algorithm performance in the test set.

A formal assessment of the radiomic pipeline’s compliance with METRICS and CLEAR guidelines was performed, and corresponding checklists are provided in Supplementary Materials S2 and S3, respectively.

### 3.3. Radiological Evaluation

**Figure 5:**
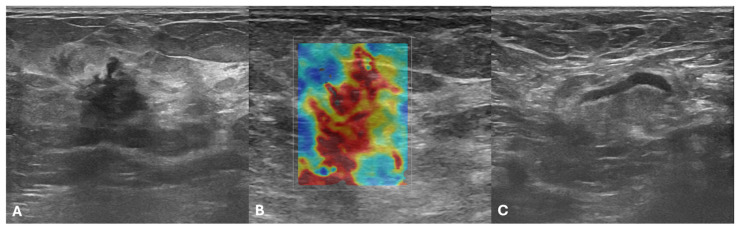
Example of a discordant case between the radiologist (False Negative) and Simple Logistic Classifier (True Positive). A 66-year-old female patient with an invasive no-special-type breast cancer (G2; ER: 90%; PgR: 30%; Ki67: 40%; Her2 1+) of the left breast. B-mode (**A**) and Shear-wave elastography (**B**) images of the primary breast lesion; (**C**) B-mode image of the ipsilateral axilla. The axillary lymph node was classified as negative by the radiologist and positive by the Simple Logistic classifier. The sentinel lymph node was found positive at histopathological analysis, and a total of five metastatic lymph nodes were detected after axillary dissection.

**Figure 6:**
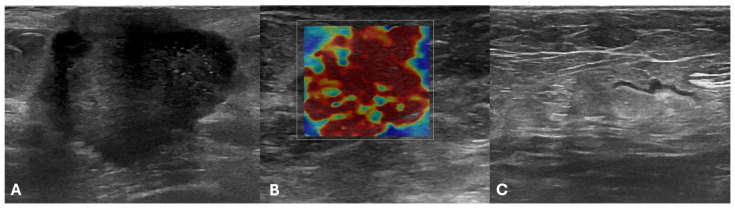
Example of a discordant case between the radiologist (True Negative) and Simple Logistic Classifier (False Positive). A 70-year-old male patient with an invasive no-special-type breast cancer (G2; ER: 90%; PgR: 30%; Ki67: 15%; Her2 1+) of the left breast. B-mode (**A**) and Shear-wave elastography (**B**) images of the primary breast lesion; (**C**) B-mode image of the ipsilateral axilla. The axillary lymph node was classified as negative by the radiologist and positive by the Simple Logistic classifier. Sentinel lymph node biopsy showed no axillary lymph node metastasis.

In the evaluation of ALN related to breast lesions included in the test set, the performance of the expert radiologist was higher than that of the ML classifier (AUC = 0.817 vs. 0.688) with accuracy of 82% (95% CI: 67–92%), sensitivity of 80% (95% CI: 56–94%), specificity of 83% (95% CI: 63–95%), positive likelihood ratio of 4.8 (95% CI: 1.9–12), negative likelihood ratio of 0.24 (95% CI: 0.1–0.6). The performance was not significantly different using McNemar’s test (*p* = 0.481). Two examples of discordant cases between the radiologist and Simple Logistic Classifier are illustrated in Figure 5 and Figure 6. In detail, in the first case, the axillary lymph node was misclassified as negative by the radiologist and correctly classified as positive by the Simple Logistic classifier; in the second case, the axillary lymph node was correctly classified as negative by the radiologist and misclassified as positive by the Simple Logistic classifier.

**Figure 7:**
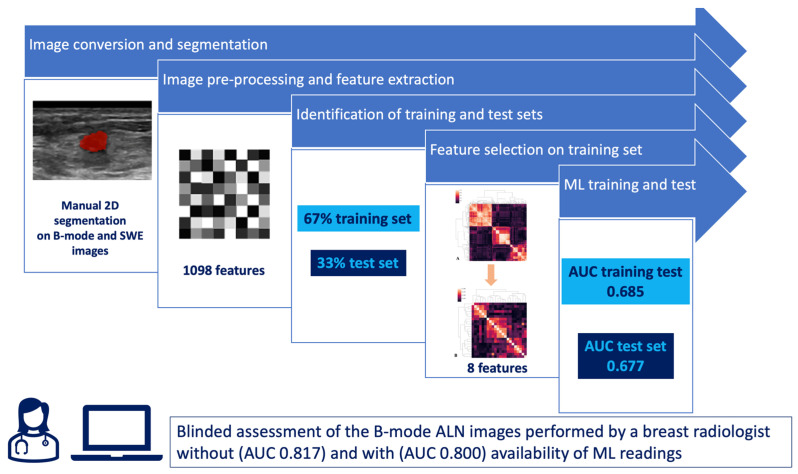
Flow diagram reporting the development of the ML model and the independent radiological assessment.

When provided with ML scores, the accuracy of the radiologist decreased from 82% to 80% (95% CI: 65–90%), but not significantly (*p* = 1.00), showing sensitivity of 80% (95% CI: 56–94%), specificity of 79% (95% CI: 58–93%), positive likelihood ratio of 3.8 (95% CI: 1.7–8.7), negative likelihood ratio of 0.25 (95% CI: 0.1–0.6). Accuracy metrics of the Simple Logistic Algorithm and the radiologist without and with availability of Simple Logistic Algorithm readings are summarized in Table 3. A flow diagram of the study is illustrated in Figure 7.

## 4. Discussion

A non-invasive ML approach integrating US and SWE was developed to preoperatively define ALN status in BC patients. This imaging modality was chosen because it is currently reported as the most sensitive for assessing ALN status, so that an additional US-derived AI tool could easily help radiologists define the axillary status. The Simple Logistic algorithm applied to radiomics features extracted from B-mode and SWE images yielded AUC values of 0.685 and 0.677, and MCC values of 0.387 and 0.375, in the training and test sets, respectively. Although the accuracy of the model was consistent between training and test sets, its performance was lower than that of an expert radiologist (accuracy: 82% vs. 68%; sensitivity: 80% vs. 75%; specificity: 83% vs. 63%) who blindly assessed US features of ALN of the test set. However, the difference in diagnostic performance was not statistically significant (*p* = 0.481). It is worth noting that a methodological discrepancy exists between the assessment performed by the ML classifier, based on radiomics features of the primary tumor lesions, and that of the radiologist, who assessed US features of the ALN. Overall, the classifier’s limited accuracy may be explained by the small sample size, monocentric study design, and the wide variability in features extracted from US images, which is influenced by acquisition parameters and operator dependence. Furthermore, the limits of manual segmentation on relatively low-resolution images may also contribute to the model’s poor performance. Rigorous pre-processing and feature selection did not allow us to overcome the abovementioned limitations. However, a remarkable finding of our investigation is the selection and inclusion of radiomics features derived from SWE in the ML model, suggesting a possible role for this imaging modality in future AI investigations applied to US breast imaging. Indeed, SWE images may be suitable, due to their intrinsic nature, for the extraction of quantitative data; additionally, lesion stiffness is reported to be correlated with lesion behavior and, possibly, with its lymphatic spread [12,13,30,31,32].

The role of AI applied to US images in predicting ALN status in BC patients has been widely explored, using B-mode images with consistent results [19,33,34,35]. Zhou et al. built a predictive model based on nine of 860 radiomics features extracted from B-mode images of breast lesions, achieving diagnostic performance better than that of a radiologist in the training set (AUC: 0.85 vs. 0.59; *p* < 0.01. More recently, Dong et al. developed a Dual-modality Complementary deep learning Network (DCFAN) that integrates B-mode US and SWE images of the primary breast lesion to differentiate benign from malignant lesions and to predict ALN status in patients with BC. In detail, DCFAN achieved an accuracy of 94.36% with an AUC of 0.97 in the classification of breast lesions, while an accuracy of 91.70% with an AUC of 0.83 in the prediction of ALN metastasis, outperforming better than radiologist [35]. Unlike the authors, we chose to explore the applicability of ML given our small datasets and to fully assess the contribution of SWE-derived radiomics features, as shown by the results of the feature-selection process. When clinical and pathological data are integrated with US radiomics features to build predictive models for ALN staging, the results are very promising [34,36]. Bove et al. have designed and compared different ML models through a Support Vector Machine (SVM) classifier, integrating clinical and pathological data (such as patient age, lesion size, grading, and molecular subtypes) and/or radiomics features extracted from the ROI of breast lesion and/or peri-tumoral tissue. The predictive model, which included clinical and pathological data and radiomic features extracted from the primary lesion and peri-tumoral tissue, showed the best diagnostic accuracy (82.1%), with an AUC of 0.886, a sensitivity of 80%, and a specificity of 65.2% [34]. These results confirm the crucial role of peritumoral tissue, the site of tumor proliferation and neoangiogenesis, in the assessment of ALN status. Unfortunately, due to the retrospective nature of our investigation, we were unable to explore the role of peritumoral tissue. Deep-learning predictive models based on clinical data and radiomics features extracted from B-mode and SWE images were also highly performing, as were radiomics-based nomograms [20,37]. Jiang et al. investigated the potential role of SWE in ALN status prediction, building a nomogram based on radiomics features extracted from SWE images, molecular subtypes, and ALN evaluation performed by a radiologist [20]. This predictive model showed good diagnostic performance in both the training set (overall C-index: 0.842; 95%CI, 0.773–0.879) and the test set (overall C-index: 0.822; 95%CI, 0.765–0.838). Furthermore, it was able to discriminate patients with low (N+ 1–2) and high (N+ > 3) ALN tumor load with good accuracy in the training (C-index: 0.827; 95%CI, 0.742–0.913) and validation (C-index: 0.810; 95%CI, 0.755–0.864) sets. However, predictive models that include pathological data imply the biopsy of the primary lesion, which is invasive and would therefore delay the use of the predictive tool in the clinical setting. Along with the use of ML and deep learning algorithms, nomograms based on radiomics features extracted from breast lesions, integrated or not with clinical data, have been developed [38,39]. Yu et al. built a radiomics nomogram, including tumor size, US-reported ALN status, and US radiomics signature of the primary lesion, to predict ALN status in patients with early-stage invasive breast cancer, achieving good performance (AUCs of 0.84 and 0.81 in the test and validation sets, respectively) [38].

Based on the already-published studies, the added value of the present investigation is the exploration of ML applied to US B-mode and SWE derived radiomics features. Even if deep-learning applications seem to perform better, our study demonstrated the feasibility of the ML approach and the potential contribution of SWE for the prediction of an outcome that still represents a major challenge in clinical practice, while based only on morphological assessment.

Our study presents several limitations that have to be acknowledged. The predictive performance of the proposed model is modest overall. It employs a very small sample size for a high-dimensional radiomics pipeline, which increases the potential for model instability despite feature reduction efforts, as evidenced by the wide confidence intervals. The limited number of patients may also have contributed to the lack of statistical significance in performance between the classifier and the radiologist. Additionally, its retrospective, single-center design limits the generalizability of the study results and introduces the potential for institutional and operator-dependent biases in image acquisition. The comparison of diagnostic performance between the ML algorithm and the expert radiologist was based on different information sources (primary lesion vs. axillary node imaging), which adds a layer of complexity to the interpretation of their results. Although this investigation was conducted according to the METRICS and CLEAR guidelines, validation of the model’s performance on an external dataset was not possible. Finally, ALN status was assessed dichotomously (positive versus negative), without considering the lymph node tumor load. This issue could not be further explored due to a few cases with more than one positive lymph node in the data set.

## 5. Conclusions

The built ML model included both US B-mode and SWE-derived radiomics features, showing a moderate performance in predicting ALN status, inferior, even if not significantly, to that of an expert radiologist.

Our findings support the use of SWE-derived radiomics features in the ML radiomics pipeline and, given the important role of US in defining ALN, additional investigations on larger multicentric datasets are encouraged to further reinforce the current evidence.

## Figures and Tables

**Table 1:** Histological diagnosis of BC lesions.

	*Number of Lesions (%)*
**ALN status**	
Negative	76 (57.1%)
Positive	57 (42.9%)
**Histologic subtype**	
Invasive ductal carcinoma NST *	106 (79.7%)
Invasive lobular carcinoma	18 (13.5%)
Other °	9 (6.8%)
**Molecular subtype**	
Luminal A	49 (36.8%)
Luminal B	67 (50.4%)
Her2+	1 (0.8%)
Triple negative	16 (12.0%)
**Tumor grade**	
G1	13 (9.8%)
G2	68 (51.1%)
G3	52 (39.1%)

* NST = non-special type. ° mucinous carcinoma, metaplastic carcinoma, tubular carcinoma, papillary carcinoma, Invasive apocrine carcinoma.

**Table 2:** Confusion matrix 2 × 2 of the algorithm performance in the test set and standard of reference (histological diagnosis) in the prediction of ALN status.

		ML		
		Negative ALN Status	Positive ALN Status	Total
**Standard of reference** **(Histological diagnosis)**	Negative ALN status	15	9	24
	Positive ALN status	5	15	20
	**Total**	20	24	44

**Table 3:** Accuracy metrics of Simple Logistic Algorithm and Radiologist without and with availability of Simple Logistic Algorithm.

	Simple L.	Radiologist	Radiologist + Simple Logistic Readings
**Accuracy**	68% (52–81%)	82% (67–92%)	80% (65–90%)
**Sensitivity**	75% (51–91%)	80% (56–94%)	80% (56–94%)
**Specificity**	63% (41–81%)	83% (63–95%)	79% (58–93%)
**Positive Likelihood Ratio**	2.0 (1.1–3.6)	4.8 (1.9–12)	3.8 (1.7–8.7)
**Negative Likelihood Ratio**	0.4 (0.2–0.9)	0.24 (0.1–0.6)	0.25 (0.1–0.6).

**Note:** data in brackets refer to 95% Confidence intervals.

## Data Availability

Research data are available from the Corresponding Author by reasonable request.

## Supplementary Materials

The following supporting information can be downloaded at: https://www.mdpi.com/article/10.3390/cancers18050862/s1, Supplementary materials S1: Details of the US B-mode and SWE imaging acquisition; Supplementary materials S2: METRICS checklist; Supplementary material S3: CLEAR checklist. References [21,40] are cited in the supplementary materials.

## Abbreviations

The following abbreviations are used in this manuscript:

ML	Machine learning
US	Ultrasound
SWE	Shear-wave elastography
ALN	Axillary lymph-node
BC	Breast cancer
MCC	Matthews Correlation Coefficient
SLNB	Sentinel lymph-node biopsy
ALND	Axillary lymph-node dissection
MRI	Magnetic Resonance Imaging
PET/CT	18F-FDG Positron Emission Tomography/Computed Tomography
ROI	Region of interest
AI	Artificial intelligence
CLEAR	Checklist for Laboratory Evidence and Reporting
METRICS	Minimum Information for Reporting a Radiomics Study
ICC	Intraclass correlation coefficient
SMOTE	Synthetic Minority Oversampling Technique
SVM	Support Vector Machine
