# Supplementary material for: Augmented Prediction of N Parameter in Breast Cancer: Is It Possible with Shear-Wave Elastography Ultrasound Radiomics?
Source: Cancers (Basel). 2026 Mar 7;18(5):862. doi: 10.3390/cancers18050862 (PMC12984800; doi:10.3390/cancers18050862)
Supplement: Supplementary file 1 [file cancers-18-00862-s001.zip › Imaging_acquisition_S1.pdf]

Imaging acquisition

US examinations were performed using a LOGIQ S8, GE Healthcare US scanner, employing a high-frequency linear probe (6-15 MHz) with radial, transverse, and longitudinal scans on both breasts. SWE was performed using a dedicated 9 MHz linear probe. The rectangular field-of-view (FOV) box of the SWE images was placed to include the entire lesion and the surrounding normal tissue (at least 3 mm). A colorimetric map (elastogram) superimposed on the US image in B-mode; the range of the color scale within the FOV varied from dark blue, indicating the lowest stiffness, to the red, indicating the highest stiffness. DICOM images were recorded and stored in the Institutional digital archives.
