# Supplementary material for: Augmented Prediction of N Parameter in Breast Cancer: Is It Possible with Shear-Wave Elastography Ultrasound Radiomics?
Source: Cancers (Basel). 2026 Mar 7;18(5):862. doi: 10.3390/cancers18050862 (PMC12984800; doi:10.3390/cancers18050862)
Supplement: Supplementary file 1 [file cancers-18-00862-s001.zip › Supplementary_Materials_S2.pdf]

METRICS Tool v1.0

Please fill out all conditions first for relevant sections and then all active items to calculate METRICS score.

Please note that default option is "No".

? Stands for explanation of items and conditions.

C Stands for conditional items or sections.

Items/Conditions	Definitions	Weights	Options
**Study Design**			
Item#1	? Adherence to radiomics and/or machine learning-specific checklists or guidelines	0.0368	◉ Yes ○ No
Item#2	? Eligibility criteria that describe a representative study population	0.0735	◉ Yes ○ No
Item#3	? High-quality reference standard with a clear definition	0.0919	◉ Yes ○ No
**Imaging Data**			
Item#4	? Multi-center	0.0438	○ Yes ◉ No
Item#5	? Clinical translatability of the imaging data source for radiomics analysis	0.0292	○ Yes ◉ No
Item#6	? Imaging protocol with acquisition parameters	0.0438	◉ Yes ○ No
Item#7	? The interval between imaging used and reference standard	0.0292	○ Yes ◉ No
**Segmentation** C			
Condition#1	? Does the study include segmentation?		◉ Yes ○ No
Condition#2	? Does the study include fully automated segmentation?		○ Yes ◉ No
Item#8	? Transparent description of segmentation methodology	0.0337	◉ Yes ○ No
Item#9	? Formal evaluation of fully automated segmentation C	0.0225	○ Yes ○ No
Item#10	? Test set segmentation masks produced by a single reader or automated tool	0.0112	◉ Yes ○ No
**Image Processing and Feature Extraction**			

Condition#3	? Does the study include hand-crafted feature extraction?		● Yes	○ No
Item#11	? Appropriate use of image preprocessing techniques with transparent description	0.0622	● Yes	○ No
Item#12	? Use of standardized feature extraction software C	0.0311	● Yes	○ No
Item#13	? Transparent reporting of feature extraction parameters, otherwise providing a default configuration statement	0.0415	● Yes	○ No

Feature Processing

Condition#4	? Does the study include tabular data?		● Yes	○ No
Condition#5	? Does the study include end-to-end deep learning?		○ Yes	● No
Item#14	? Removal of non-robust features C	0.0200	● Yes	○ No
Item#15	? Removal of redundant features C	0.0200	● Yes	○ No
Item#16	? Appropriateness of dimensionality compared to data size C	0.0300	○ Yes	● No
Item#17	? Robustness assessment of end-to-end deep learning pipelines C	0.0200	○ Yes (disabled)	○ No (disabled)

Preparation for Modeling

Item#18	? Proper data partitioning process	0.0599	● Yes	○ No
Item#19	? Handling of confounding factors	0.0300	○ Yes	● No

Metrics and Comparison

Item#20	? Use of appropriate performance evaluation metrics for task	0.0352	● Yes	○ No
Item#21	? Consideration of uncertainty	0.0234	● Yes	○ No
Item#22	? Calibration assessment	0.0176	○ Yes	● No
Item#23	? Use of uni-parametric imaging or proof of its inferiority	0.0117	○ Yes	● No
Item#24	? Comparison with a non-radiomic approach or proof of added clinical value	0.0293	● Yes	○ No
Item#25	? Comparison with simple or classical statistical models	0.0176	○ Yes	● No

Testing

Item#26	?	Internal testing	0.0375	◉ Yes	○ No
Item#27	?	External testing	0.0749	○ Yes	◉ No
**Open Science**					
Item#28	?	Data availability	0.0075	○ Yes	◉ No
Item#29	?	Code availability	0.0075	○ Yes	◉ No
Item#30	?	Model availability	0.0075	○ Yes	◉ No
			**Total METRICS score:**	**68.0%**	
		?	**Quality category:**	**Good**	
		?	**Publication ID:**		

If you publish any work which uses this tool, please cite the following publication:

Kocak B, Akinci D'Antonoli T, Mercaldo N, et al. METhodological RadiomICs Score (METRICS): a quality scoring tool for radiomics research endorsed by EuSoMII. Insights Imaging. 2024;15(1):8. Published 2024 Jan 17. doi:10.1186/s13244-023-01572-w
